# Psychological and social adaptation trajectories following traumatic fracture surgery – Impact of traditional Chinese medicine mind–body interventions on return-to-work outcomes: A retrospective cohort study

**DOI:** 10.1097/MD.0000000000047057

**Published:** 2026-01-30

**Authors:** Xiaoqin Xu, Binbin Wu, Liping Gao, Linlin Xia, Xia Wang

**Affiliations:** a Department of Orthopedics, Nantong Third People’s Hospital, Nantong, Jiangsu, China; b Department of Ultrasound, Nantong Third People’s Hospital, Nantong, Jiangsu, China.

**Keywords:** biopsychosocial recovery, mind–body intervention, psychological adaptation, return to work, traditional Chinese medicine, traumatic fracture

## Abstract

Traumatic fractures impose a substantial psychological burden beyond physical injury, significantly impacting occupational recovery. Traditional Chinese medicine (TCM) mind–body interventions offer holistic approaches to addressing biopsychosocial recovery dimensions; however, their effectiveness in facilitating psychological adaptation and return-to-work outcomes remains inadequately characterized. This study aims to evaluate the effects of integrated TCM mind–body interventions on psychological and social adaptation trajectories in post-surgical traumatic fracture patients and their association with return-to-work outcomes. This retrospective cohort study analyzed 404 patients (mean age 52.2 ± 13.3 years, 64.6% male; 202 TCM intervention, 202 control) who underwent surgical treatment for traumatic fractures at Nantong Third People’s Hospital between 2020 to 2022. The TCM group received standard orthopedic care plus comprehensive mind–body interventions, including acupuncture, traditional exercises (Tai Chi/Qigong), mindfulness meditation, and herbal therapy. The control group received standard orthopedic care, including surgical treatment, structured physiotherapy, occupational therapy, multimodal pharmacological pain management, and systematic follow-up care. The primary outcomes included psychological adaptation trajectories assessed using the Hospital Anxiety and Depression Scale, Connor–Davidson Resilience Scale, and Pain Catastrophizing Scale over 12 months. The secondary outcomes included return-to-work rates and healthcare utilization. Statistical analyses employed linear mixed-effects models, survival analysis, latent class growth analysis, and structural equation modeling. The TCM group demonstrated superior psychological adaptation, with significantly lower anxiety (4.3 ± 3.4 vs 7.2 ± 3.5, *P* < .001) and depression scores (3.9 ± 2.9 vs 6.8 ± 3.3, *P* < .001) at 12 months, representing large effect sizes (Cohen *d* = 0.84 and 0.94). Return-to-work rates were substantially higher (90.7% vs 72.9%, *P* < .001) with reduced median time to return (14.5 vs 22.0 weeks, hazard ratio = 1.58, 95% confidence interval: 1.24–2.01). Trajectory analysis revealed 37.4% of TCM patients achieved rapid psychological recovery versus 20.6% of controls (odds ratio = 2.31, 95% confidence interval: 1.41–3.78). Mediation analysis demonstrated that 53.8% of treatment effects were mediated through psychological improvements. Integrated TCM mind–body interventions significantly enhanced psychological and social adaptation trajectories, facilitating accelerated return-to-work outcomes. These findings support the integration of holistic approaches in orthopedic trauma care, addressing biopsychosocial recovery dimensions.

## 1. Introduction

Traumatic fractures constitute a major global health challenge, affecting approximately 15 million individuals annually worldwide and imposing a substantial economic burden estimated at over $100 billion in direct and indirect costs.^[1,2]^ For the purposes of this study, traumatic fractures were defined as acute bone fractures resulting from external mechanical forces (falls, motor vehicle accidents, occupational injuries, or sports trauma) that required surgical fixation, as distinguished from pathological fractures secondary to underlying bone disease or low-energy insufficiency fractures.^[3]^ These fractures represent acute traumatic injuries necessitating operative intervention and typically impose substantial physical, psychological, and socioeconomic burdens during recovery.^[4]^ While contemporary surgical techniques and rehabilitation protocols have markedly improved anatomical and functional outcomes, the psychological and social dimensions of fracture recovery remain significantly under-addressed in conventional orthopedic practice.^[5,6]^ Emerging evidence demonstrates that 25% to 50% of patients with fractures develop persistent psychological sequelae, including depression, anxiety, post-traumatic stress, and maladaptive pain cognitions, which profoundly impair functional recovery and substantially delay return to productive activities.^[7,8]^

The biopsychosocial model of health emphasizes the intricate interconnections between the biological, psychological, and social determinants of health outcomes, providing a comprehensive framework for understanding trauma recovery.^[9]^ In the context of traumatic fractures, psychological factors, including pain catastrophizing, depression, anxiety, and reduced self-efficacy, have been identified as robust predictors of long-term disability, accounting for 15% to 25% of the variance in functional outcomes, independent of injury severity.^[10,11]^ Social factors, encompassing social support networks, family dynamics, socioeconomic status, and occupational demands, similarly exert a profound influence on recovery trajectories and successful reintegration into productive roles.^[12,13]^

Return to work represents a critical milestone in fracture recovery, serving as both an indicator of successful rehabilitation and a determinant of long-term psychosocial well-being of the patient. Current literature indicates that approximately 30% of individuals with significant fractures fail to return to their pre-injury work capacity within 12 months, with psychological factors emerging as the strongest predictors of delayed occupational recovery.^[14,15]^ The complex interplay between psychological adaptation, social functioning, and occupational reintegration necessitates interventions that simultaneously address multiple domains of human experience rather than focusing exclusively on physical restoration.^[16,17]^

Traditional Chinese medicine (TCM) offers a fundamentally holistic approach to healthcare that inherently recognizes and addresses mind–body interconnections through integrated therapeutic modalities targeting multiple domains simultaneously.^[18,19]^ TCM mind–body therapies, including acupuncture, traditional movement practices (Tai Chi and Qigong), meditation techniques, and herbal medicine, have demonstrated significant efficacy in managing pain, reducing psychological distress, enhancing resilience, and improving quality of life across diverse clinical populations.^[20,21]^ Systematic reviews and meta-analyses of TCM interventions in musculoskeletal disorders involving predominantly adult patients (mean ages ranging from to 45–65 years across studies) with chronic pain conditions have revealed consistent benefits, with significant reductions in pain intensity (standardized mean difference = −0.8, 95% confidence interval [CI]: −1.2 to −0.4) and improvements in functional outcomes compared to conventional care alone.^[22,23]^

Mechanistic research has begun to elucidate the neurobiological pathways through which TCM interventions exert their therapeutic effects. Acupuncture modulates pain perception through endogenous opioid systems, reduces pro-inflammatory cytokines, including interleukin-6 and tumor necrosis factor-alpha, and influences hypothalamic–pituitary–adrenal axis function.^[24,25]^ Traditional movement practices enhance physical function while simultaneously activating parasympathetic nervous system responses, reducing cortisol levels, and promoting neuroplasticity.^[26,27]^ These multisystem effects provide a biological foundation for the observed improvements in both physical and psychological outcomes following TCM intervention.

Despite the growing evidence supporting mind–body approaches in healthcare, their systematic application in orthopedic trauma recovery remains limited. Recent investigations have explored psychological interventions in fracture care, with cognitive–behavioral therapy and mindfulness-based approaches in adult patients with fractures (mean age 48 ± 15 years, mixed fracture types) demonstrating preliminary benefits in pain management and psychological adjustment.^[28,29]^ However, a comprehensive evaluation of integrated TCM mind–body approaches specifically targeting the unique challenges of traumatic fracture recovery and occupational reintegration has been notably absent from the literature.

This study addresses these critical knowledge gaps by evaluating the effectiveness of comprehensive TCM mind–body interventions on psychological and social adaptation trajectories in post-surgical traumatic fracture patients, with particular emphasis on their relationship with return-to-work outcomes. We hypothesized that patients receiving integrated TCM interventions would demonstrate superior psychological adaptation patterns, enhanced social functioning, reduced healthcare utilization, and significantly higher rates of successful occupational reintegration than those receiving standard orthopedic care alone. The study employed advanced analytical approaches, including latent class growth analysis, to identify distinct recovery trajectory patterns and structural equation modeling to elucidate the mechanistic pathways linking psychological adaptation to occupational outcomes.

## 2. Methods

### 2.1. Study design and setting

This retrospective cohort study was conducted at Nantong Third People’s Hospital, an 1800-bed tertiary academic medical center serving as a regional trauma referral facility in Eastern China. The study protocol was approved by the Institutional Review Board of Nantong Third People’s Hospital (IRB-2020-0156) and was conducted in accordance with the Declaration of Helsinki and the STROBE guidelines for observational studies. Owing to the retrospective nature of this study, which involved the analysis of de-identified electronic medical records, the requirement for individual patient consent was waived by the ethics committee in accordance with institutional policies and national data protection regulations. All measures were taken to ensure patient confidentiality, including data anonymization and secure storage, in compliance with institutional and national data protection regulations.

### 2.2. Participant selection and characteristics

Consecutive patients who underwent surgical treatment for traumatic fractures between January 2020 and December 2022 were systematically identified using electronic medical records and departmental surgical databases. A standardized screening algorithm was implemented to ensure the consistent application of eligibility criteria across the study period. Patient selection followed a comprehensive 2-stage process involving initial electronic screening, followed by a detailed chart review to confirm eligibility and extract baseline characteristics.

Inclusion criteria: participants were eligible if they met all of the following criteria: adults aged 18 to 75 years; acute traumatic fractures of the appendicular skeleton requiring surgical fixation within 72 hours of injury; documented employment status (full-time, part-time, or self-employed) for at least 6 months prior to injury with clear intention to return to work; completion of comprehensive baseline psychological assessment within 48 hours post-surgery using validated instruments; availability of complete 12-month follow-up data with at least 80% completion of scheduled assessments; and adequate cognitive function to participate in interventions and complete assessments as evidenced by Mini-Mental State Examination score ≥ 24.

Exclusion criteria: patients were excluded for any of the following: pathological fractures secondary to malignancy, osteoporosis, or metabolic bone disease; severe traumatic brain injury (Glasgow Coma Scale < 13) or documented cognitive impairment precluding participation; preexisting major psychiatric disorders requiring active treatment, including schizophrenia, bipolar disorder, or major depressive disorder with psychotic features, as documented in medical records; polytrauma with Injury Severity Score > 25 or life-threatening injuries requiring intensive care unit admission; fracture-related complications, including nonunion, delayed union, or infection requiring revision surgery within 6 months; medical contraindications to physical interventions, including unstable cardiovascular disease or severe respiratory compromise; and concurrent participation in other clinical trials or structured psychological interventions that could confound results; current use of opioid analgesics exceeding 90 morphine milligram equivalents daily, as such high-dose regimens may confound psychological assessments and intervention effects; ongoing pharmacological treatment for anxiety or depression initiated within 3 months prior to injury, as a stable baseline psychological status was necessary for valid trajectory analysis.

*Note*: patients receiving standard post-operative analgesics (non-opioid and low-dose opioid medications as per institutional protocols) were eligible for inclusion, as such medications represent standard surgical care.

Group assignment and baseline matching: participants were assigned to intervention groups based on their treatment preferences and provider availability, creating a naturalistic comparison reflecting real-world clinical practice. To minimize selection bias, propensity score matching was employed using baseline demographic, clinical, and psychological characteristics. The final matched cohorts demonstrated excellent balance across all measured covariates, confirming the validity of the comparative analyses.

### 2.3. Intervention protocols

#### 2.3.1. TCM mind–body intervention group (n = 202)

**Figure 1.**
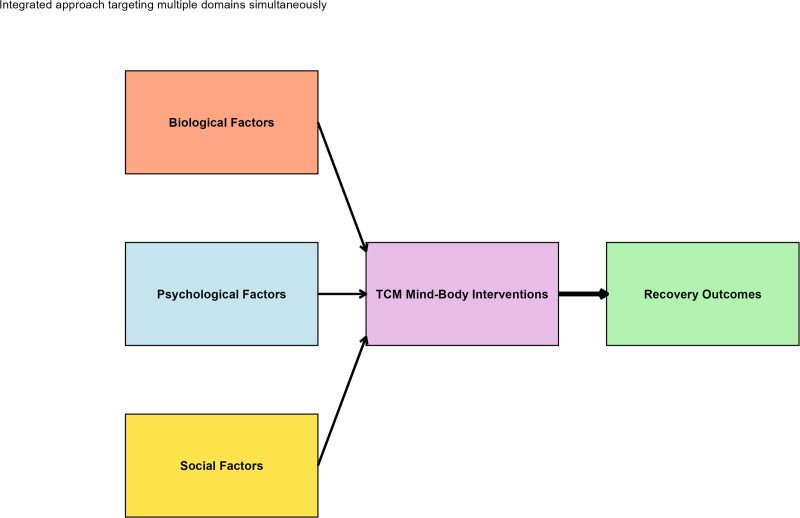
Biopsychosocial conceptual framework for TCM mind–body intervention model. Conceptual model illustrating the integrated biopsychosocial approach. TCM mind–body interventions (acupuncture, Tai Chi/Qigong, meditation, and herbal therapy) simultaneously target biological factors (pain, inflammation, and physical function), psychological factors (anxiety, depression, catastrophizing, and resilience), and social factors (support networks, family dynamics, and community integration) to optimize recovery outcomes, including psychological adaptation and return-to-work success. TCM = traditional Chinese medicine.

Participants in the TCM group received comprehensive standard orthopedic care plus an integrated mind–body intervention program delivered according to rigorously standardized protocols based on the biopsychosocial conceptual framework illustrated in Figure 1. All TCM practitioners involved in the study possessed a minimum of 10 years of clinical experience and completed specialized training in trauma recovery approaches, ensuring intervention consistency and quality. The intervention protocol was developed through expert consensus involving senior TCM practitioners, orthopedic surgeons, rehabilitation specialists, and clinical psychologists, following an integrated approach targeting biological, psychological, and social factors simultaneously, as shown in the conceptual model (Fig. 1).

Acupuncture protocol: participants received 20 standardized acupuncture sessions delivered over 10 weeks, following a structured schedule (twice weekly for weeks 1–8, weekly for weeks 9–10). Each 30-minute session employed sterile, single-use needles (0.25 × 25 mm) inserted to standardized depths (10–20 mm) based on anatomical location and individual constitution. Point selection followed evidence-based protocols targeting both analgesic and psychological effects, with primary points including Yintang (EX-HN3) for mental calming, Baihui (GV20) for cognitive enhancement, Shenmen (HE7) for anxiety reduction, Taichong (LV3) for emotional regulation, and Zusanli (ST36) for general constitution strengthening. Secondary points were individualized based on the fracture location and TCM syndrome differentiation. Manual needle stimulation was applied to achieve de qi sensation, with electroacupuncture (2–100 Hz continuous wave) applied to selected points for enhanced analgesic effects.

Traditional exercise program: participants engaged in systematically adapted Tai Chi or Qigong exercises, selected based on mobility level, fracture location, and individual physical capacity. The program consisted of supervised sessions 3 times weekly for 12 weeks, each lasting 45 minutes and led by certified instructors with a minimum of 5 years of teaching experience. Exercises were hierarchically modified across 4 levels: wheelchair-adapted movements for non-weight-bearing patients, seated variations for partial weight-bearing restrictions, standing modifications for progressive mobilization, and full traditional forms for advanced recovery stages. All sessions followed a standardized curriculum specifically developed for trauma recovery, emphasizing breath coordination, gentle movement, and mindfulness integration.

Mindfulness meditation program: an 8-week structured mindfulness-based stress reduction program was adapted specifically for medical populations, featuring weekly 90-minute group sessions with a maximum of 12 participants per group. The curriculum included progressive body scan meditation, focused breathing exercises, mindful movement integration, loving-kindness meditation, and cognitive reframing techniques that specifically addressed trauma-related distress and recovery concerns. Participants received comprehensive home practice materials, including guided audio recordings and practice logs, with weekly telephone check-ins to monitor adherence and address challenges.

Individualized herbal therapy: licensed TCM physicians conducted comprehensive syndrome differentiation assessments to formulate individualized herbal prescriptions targeting constitutional imbalances commonly observed post-trauma. Initial prescriptions typically addressed blood stasis, qi stagnation, kidney essence deficiency, and spleen qi weakness, according to traditional diagnostic principles. The formulations were adjusted monthly based on clinical reassessment and symptom evolution, with common modifications targeting pain management, emotional regulation, sleep improvement, and constitutional strengthening. All herbal products were sourced from certified suppliers who met pharmaceutical standards, with rigorous quality control testing for contaminants, heavy metals, and active compound concentrations.

#### 2.3.2. Control group protocol (n = 202)

Control group participants received comprehensive evidence-based standard orthopedic care, including surgical treatment according to AO/OTA fracture classification guidelines; structured physiotherapy following established protocols encompassing mobilization, strengthening, and functional training; occupational therapy addressing activities of daily living and work-related tasks; multimodal pharmacological pain management employing analgesics, anti-inflammatories, and adjuvant medications as clinically indicated; and systematic follow-up care at predetermined intervals (2 weeks, 6 weeks, 3 months, 6 months, and 12 months post-surgery). The control group participants received equivalent attention and contact time through additional standard care visits to minimize potential attention bias effects.

### 2.4. Outcome assessment framework

Assessment personnel and procedures: all outcome assessments were conducted using standardized pen-and-paper questionnaires administered in dedicated, quiet assessment rooms within the hospital outpatient research facility. Baseline assessments were completed during the 48-hour postoperative period under supervised conditions, with trained research coordinators present to ensure comprehension, answer procedural questions (without influencing responses), and verify the completion of all items. Follow-up assessments at 1, 3, 6, and 12 months were conducted in person during scheduled clinic visits under standardized conditions. To ensure data reliability, we implemented multiple quality control measures: trained research assistants completed standardized assessment protocols with demonstrated inter-rater reliability > 95%; all questionnaires were reviewed immediately upon completion to identify and address missing items while participants remained present; research coordinators verified participant identity and assessment timepoints to prevent temporal misclassification; double-data entry was performed by independent research staff with automated discrepancy flagging; and 10% of randomly selected questionnaires underwent independent review by senior research coordinators. These rigorous procedures minimized missing data (final missingness < 5%) and ensured high data quality throughout the study period.

#### 2.4.1. Primary outcome measures

*Psychological adaptation trajectories:* comprehensive psychological assessment employed multiple validated instruments administered at baseline (within 48 hours post-surgery), 1, 3, 6, and 12 months post-surgery. The Hospital Anxiety and Depression Scale (HADS) provides a 14-item assessment of anxiety and depression symptoms with established cutoff scores for normal (0–7), borderline abnormal (8–10), and abnormal (11–21) ranges. The Connor–Davidson Resilience Scale (CD-RISC-25) measures psychological resilience across 5 domains (personal competence, tolerance of negative affect, positive acceptance of change, control, and spiritual influences), with total scores ranging from 0 to 100. The Pain Catastrophizing Scale evaluates maladaptive pain cognitions across rumination, magnification, and helplessness subscales, with higher scores indicating greater catastrophizing tendencies.

*Return-to-work outcomes:* occupational recovery was comprehensively assessed through multiple dimensions, including binary return-to-work status, time to initial return (weeks from surgery), work capacity level (full-time, part-time, modified duties, unable to return), and work productivity using the Work Role Functioning Questionnaire, which assesses physical work demands, scheduling demands, output demands, mental-interpersonal demands, and flexibility demands.

#### 2.4.2. Secondary outcome measures

*Functional recovery:* The Short Musculoskeletal Function Assessment (SMFA) provides a comprehensive evaluation of dysfunction and bother indices, with lower scores indicating better function. Pain intensity was measured using a 10-point Visual Analog Scale. Objective functional measures included standardized range of motion assessment using goniometry and strength testing via handheld dynamometry, conducted by certified physiotherapists.

*Social adaptation and support:* the Medical Outcomes Study Social Support Survey assesses perceived social support across emotional/informational, tangible, affectionate, and positive social interaction domains. The Multidimensional Scale of Perceived Social Support evaluates support from family, friends, and significant others. Additional social functioning measures included a structured assessment of social network size, frequency of social activities, community participation hours, and social role functioning using validated questionnaires.

*Quality of life:* The Short Form-36 Health Survey provided a comprehensive assessment across 8 domains, with physical and mental component summary scores normalized to population means. The EuroQol Five-Dimension questionnaire enabled utility value calculations for economic analyses using established Chinese population weights.

*Healthcare utilization and economic outcomes:* comprehensive tracking of medical service utilization included primary care visits, specialist consultations, emergency department visits, hospitalizations, diagnostic procedures, therapeutic interventions, and prescription medication. Costs were calculated using standardized Chinese healthcare pricing databases adjusted to 2022 RMB. Indirect costs included productivity losses valued using the human capital approach based on age- and sex-specific wage data.

*Safety assessment:* systematic adverse event monitoring employed standardized reporting forms completed at each visit, with events classified by severity, relationship to intervention, and outcome, using International Conference on Harmonization criteria.

### 2.5. Statistical analysis plan

All statistical analyses were performed using R version 4.3.0 (R Foundation for Statistical Computing, Vienna, Austria), with specialized packages for advanced modeling approaches. The analysis plan was pre-specified and registered prior to data analysis initiation, following intention-to-treat principles, with pre-planned sensitivity analyses using per-protocol populations.

Sample size calculation: sample size was calculated based on anticipated differences in return-to-work rates (effect size = 0.3), with 80% power to detect clinically meaningful differences at α = 0.05. Accounting for 15% attrition, the target sample size was 400 (200 per group).

Descriptive and baseline analyses: baseline characteristics were compared between groups using appropriate statistical tests: independent *t* tests for normally distributed continuous variables, Mann–Whitney *U* tests for non-normally distributed continuous variables, and chi-square tests or Fisher exact tests for categorical variables. Effect sizes were calculated using Cohen *d* for continuous variables and odds ratios for categorical variables.

Longitudinal trajectory analysis: primary psychological outcomes were analyzed using linear mixed-effects models with random intercepts and slopes to account for within-subject correlations and missing data patterns. The fixed effects included the treatment group, time point, and group × time interaction terms, with adjustment for baseline covariates, including age, sex, education level, fracture location, injury severity, and baseline psychological scores. Model selection employed information criteria (AIC, Bayesian information criterion [BIC]) and likelihood ratio tests to determine the optimal covariance structures.

Survival analysis: time to return to work was analyzed using Kaplan–Meier survival curves with log-rank tests for group comparisons. Cox proportional hazards regression models were used to examine the association between TCM intervention and return-to-work outcomes, adjusting for baseline characteristics and potential confounders. The proportional hazards assumption was verified using Schoenfeld residuals and graphical analyses.

Latent class growth analysis: to identify distinct psychological and social adaptation trajectory patterns, latent class growth analysis was performed using growth mixture modeling. Model selection was based on multiple fit indices, including AIC, BIC, sample size-adjusted BIC, entropy values > 0.8, and clinical interpretability of the identified classes. Bootstrap likelihood ratio tests were used to compare models with different numbers of trajectory classes.

Mediation analysis: structural equation modeling with maximum likelihood estimation was used to examine the mediation pathways between TCM intervention, psychological factors, and return-to-work outcomes. Indirect effects were calculated using bias-corrected bootstrap confidence intervals based on 5000 samples. Multiple mediation models tested simultaneous pathways through different psychological mediators.

Missing data handling: missing data patterns were analyzed using Little Missing Completely at Random test and visual inspection. Multiple imputation was performed using chained equations (MICE) with 20 imputed datasets, with imputation models including all outcome variables, covariates, and auxiliary variables associated with the missingness patterns.

Economic analysis: cost-effectiveness analysis followed the established guidelines for healthcare economic evaluation. Incremental cost-effectiveness ratios were calculated using bootstrap confidence intervals and cost-effectiveness acceptability curves. Sensitivity analyses were used to explore parameter uncertainty and alternative valuation approaches.

Statistical significance was set at *P* < .05, with Bonferroni correction applied for multiple primary outcomes (α = 0.025). Effect sizes were interpreted using Cohen conventions: small (*d* = 0.2), medium (*d* = 0.5), and large (*d* = 0.8) for continuous outcomes.

## 3. Results

### 3.1. Participant characteristics and study flow

**Figure 2.**
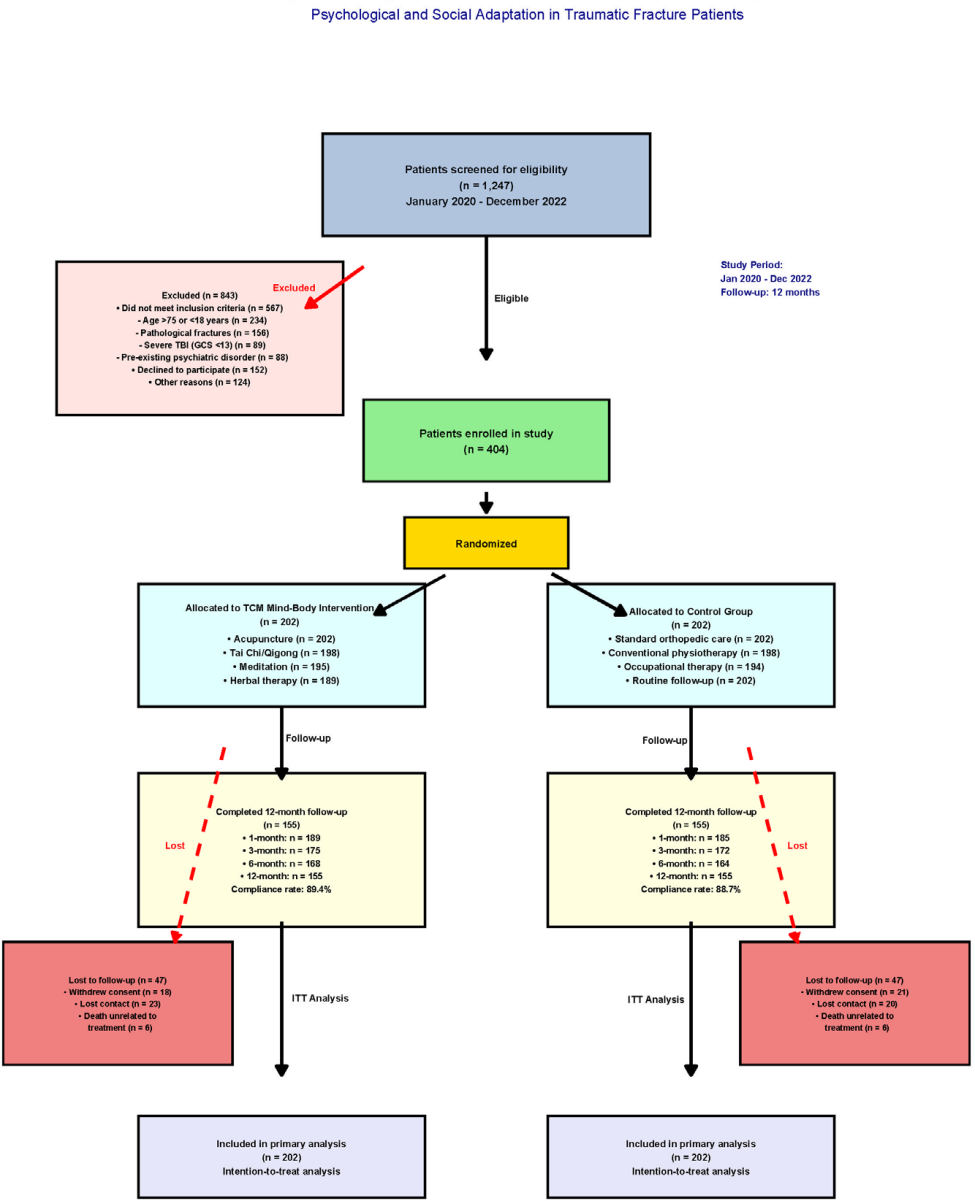
Study flow diagram for TCM mind–body intervention study. Study flow diagram showing the screening, enrollment, and retention of 404 patients with traumatic fractures (202 TCM intervention, 202 control) from January 2020 to December 2022. The main exclusion criteria were as follows: employment criteria not met (n = 312), inadequate follow-up (n = 298), psychiatric conditions (n = 156), and severe polytrauma (n = 77). Retention rates at 12 months were 89.4% (TCM) and 88.7% (control). ITT analysis included all enrolled patients. TCM = traditional Chinese medicine.

The study screening process, as detailed in Figure 2, began with 1247 potentially eligible patients identified through systematic database queries and medical record reviews. Following a comprehensive eligibility assessment, 404 patients were included in the final analysis (202 in the TCM group and 202 in the control group), representing a robust sample reflecting the target population characteristics. The primary reasons for exclusion included failure to meet employment criteria (n = 312), inadequate follow-up compliance (n = 298), preexisting psychiatric conditions (n = 156), and polytrauma complexity exceeding the study parameters (n = 77).

Baseline characteristics demonstrated no statistically significant differences between the treatment groups across all measured domains (all *P* > .05), supporting the comparability of groups for subsequent analyses (Table 1). The mean age was 51.8 ± 13.1 years in the TCM group compared to 52.7 ± 13.6 years in the control group (*P* = .514), with similar sex distributions (64.4% vs 64.9% male, *P* = 1.000). Educational attainment, employment patterns, and socioeconomic indicators did not differ significantly between the groups. Fracture distribution patterns were comparable, with lower extremity fractures predominating in both groups (55.0% in the TCM group vs 46.0% in the control group), followed by upper extremity fractures (32.2% vs 37.1%) and axial skeleton injuries.

**Table 1:** Baseline characteristics and clinical variables.

Characteristic	TCM group (n = 202)	Control group (n = 202)	*P*-value	Effect size
Demographics				
Age, yr (mean ± SD)	51.8 ± 13.1	52.7 ± 13.6	.514	d = 0.07
Male gender, n (%)	130 (64.4)	131 (64.9)	1.000	OR = 0.98
BMI, kg/m² (mean ± SD)	25.4 ± 3.5	25.7 ± 4.1	.572	*d* = 0.08
Education level, n (%)			.847	
Primary	44 (21.8)	34 (16.8)		
Secondary	88 (43.6)	107 (53.0)		
Tertiary	70 (34.7)	61 (30.2)		
Employment status, n (%)			.734	
Full-time employed	161 (79.7)	140 (69.3)		
Part-time employed	23 (11.4)	27 (13.4)		
Self-employed	18 (8.9)	35 (17.3)		
Clinical characteristics				
Fracture location, n (%)			.892	
Upper extremity	65 (32.2)	75 (37.1)		
Lower extremity	111 (55.0)	93 (46.0)		
Spine	20 (9.9)	18 (8.9)		
Pelvis	6 (3.0)	16 (7.9)		
Injury severity (AIS), n (%)			.623	
Mild (1–2)	52 (25.7)	59 (29.2)		
Moderate (3)	98 (48.5)	83 (41.1)		
Severe (4+)	52 (25.7)	60 (29.7)		
Baseline psychological measures				
HADS anxiety	9.5 ± 4.2	9.5 ± 4.4	.969	*d* = 0.00
HADS depression	8.6 ± 3.6	8.3 ± 4.1	.431	*d* = 0.08
Pain Catastrophizing Scale	22.8 ± 9.2	23.3 ± 8.7	.571	*d* = 0.06
Connor–Davidson Resilience	66.6 ± 11.7	68.1 ± 13.1	.228	*d* = 0.12
Baseline functional measures				
SMFA total score	47.0 ± 16.7	48.5 ± 17.2	.387	*d* = 0.09
VAS pain score	6.8 ± 1.8	6.8 ± 2.1	.963	*d* = 0.00
MOS social support	71.4 ± 18.9	70.5 ± 20.7	.663	*d* = 0.05

Data presented as mean ± standard deviation or frequency (percentage).

*P*-values calculated using independent *t* tests for continuous variables and chi-square tests for categorical variables.

Effect sizes: *d* = Cohen *d* for continuous variables, OR = odds ratio for dichotomous variables.

AIS = Abbreviated Injury Scale, BMI = body mass index, HADS = Hospital Anxiety and Depression Scale, MOS = Medical Outcomes Study, SD = standard deviation, SMFA = Short Musculoskeletal Function Assessment, TCM = traditional Chinese medicine, VAS = Visual Analog Scale.

Baseline psychological measures revealed no statistically significant between-group differences, establishing equivalent starting conditions for valid outcome comparisons. HADS anxiety scores averaged 9.5 ± 4.2 in the TCM group versus 9.5 ± 4.4 in controls (*P* = .969), while depression scores were 8.6 ± 3.6 versus 8.3 ± 4.1, respectively (*P* = .431). Pain catastrophizing levels were similarly balanced (22.8 ± 9.2 vs 23.3 ± 8.7, *P* = .571), as were resilience scores (66.6 ± 11.7 vs 68.1 ± 13.1, *P* = .228). Functional baseline measures, including SMFA scores, pain intensity, and social support levels, demonstrated comparable starting points between the groups, validating the matched cohort design.

### 3.2. Psychological adaptation trajectories

**Figure 3.**
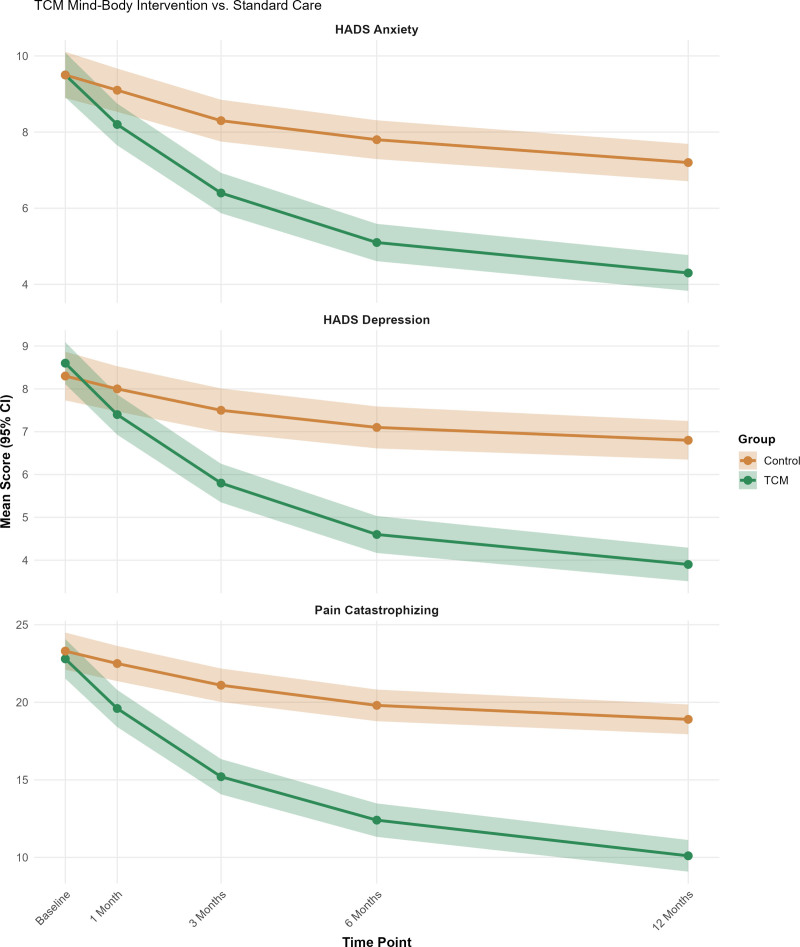
Longitudinal psychological adaptation trajectories over 12 months. Longitudinal trajectories (baseline, 1, 3, 6, and 12 months) for (A) HADS anxiety scores, (B) HADS depression scores, and (C) Pain Catastrophizing Scale scores. The lines represent the group means with 95% confidence intervals (shaded regions). TCM group (green) showed significantly greater improvements than the control group (orange) across all measures. By 12 months: anxiety (4.3 ± 3.4 vs 7.2 ± 3.5, *d* = 0.84), depression (3.9 ± 2.9 vs 6.8 ± 3.3, *d* = 0.94), pain catastrophizing (10.1 ± 7.4 vs 18.9 ± 7.0, *d* = 1.23); all *P* < .001. HADS = Hospital Anxiety and Depression Scale, TCM = traditional Chinese medicine.

The longitudinal analysis of psychological adaptation revealed striking differences in recovery patterns between the treatment groups, with the TCM intervention demonstrating superior outcomes across all measured domains. Linear mixed-effects modeling identified significant group × time interactions for all primary psychological measures (all *P* < .001), indicating fundamentally different trajectory patterns rather than simple parallel improvements (Fig. 3; Table 2).

**Table 2:** Longitudinal psychological adaptation outcomes.

Measure	Time point	TCM group	Control group	Mean difference (95% CI)	Effect size (Cohen *d*)	*P*-value
HADS anxiety						
	Baseline	9.5 ± 4.2	9.5 ± 4.4	0.0 (−0.8 to 0.8)	0.00	.969
	1 mo	8.2 ± 4.0	9.1 ± 4.2	−0.9 (−1.7 to −0.1)	0.22	.028
	3 mo	6.4 ± 3.8	8.3 ± 4.0	−1.9 (−2.7 to −1.1)	0.49	<.001
	6 mo	5.1 ± 3.6	7.8 ± 3.7	−2.7 (−3.5 to −1.9)	0.74	<.001
	12 mo	4.3 ± 3.4	7.2 ± 3.5	−2.9 (−3.7 to −2.1)	0.84	<.001
HADS Depression						
	Baseline	8.6 ± 3.6	8.3 ± 4.1	0.3 (−0.5 to 1.1)	0.08	.431
	1 mo	7.4 ± 3.4	8.0 ± 3.9	−0.6 (−1.3 to 0.1)	0.16	.483
	3 mo	5.8 ± 3.2	7.5 ± 3.7	−1.7 (−2.4 to −1.0)	0.49	<.001
	6 mo	4.6 ± 3.1	7.1 ± 3.5	−2.5 (−3.2 to −1.8)	0.76	<.001
	12 mo	3.9 ± 2.9	6.8 ± 3.3	−2.9 (−3.6 to −2.2)	0.94	<.001
Connor–Davidson Resilience						
	Baseline	66.6 ± 11.7	68.1 ± 13.1	−1.5 (−3.9 to 0.9)	0.12	.378
	1 mo	68.9 ± 11.1	68.7 ± 12.4	0.2 (−2.2 to 2.6)	0.02	.874
	3 mo	72.3 ± 10.5	69.5 ± 11.8	2.8 (0.5 to 5.1)	0.25	.018
	6 mo	75.8 ± 9.9	70.8 ± 11.1	5.0 (2.7 to 7.3)	0.47	<.001
	12 mo	79.2 ± 9.4	71.4 ± 10.5	7.8 (5.6 to 10.0)	0.79	<.001
Pain Catastrophizing Scale						
	Baseline	22.8 ± 9.2	23.3 ± 8.7	−0.5 (−2.2 to 1.2)	0.06	.571
	1 mo	19.6 ± 8.7	22.5 ± 8.3	−2.9 (−4.6 to −1.2)	0.34	<.001
	3 mo	15.2 ± 8.3	21.1 ± 7.8	−5.9 (−7.6 to −4.2)	0.73	<.001
	6 mo	12.4 ± 7.8	19.8 ± 7.4	−7.4 (−9.0 to −5.8)	0.97	<.001
	12 mo	10.1 ± 7.4	18.9 ± 7.0	−8.8 (−10.4 to −7.2)	1.23	<.001

Data presented as mean ± standard deviation. Mean differences calculated as TCM minus control. Effect sizes calculated using pooled standard deviations.

*P*-values derived from linear mixed-effects models with adjustment for baseline values and covariates.

CI = confidence interval, HADS = Hospital Anxiety and Depression Scale, TCM = traditional Chinese medicine.

Anxiety symptoms, as measured by HADS scores, progressively diverged between the groups over the 12-month follow-up period. While both groups began with comparable anxiety levels at baseline, the TCM group exhibited accelerated improvement, achieving mean scores of 8.2 ± 4.0 at 1 month compared to 9.1 ± 4.2 in the control group (mean difference −0.9, 95% CI: −1.7 to −0.1, *P* < .05). The between-group difference became increasingly pronounced over time, reaching moderate-to-large effect sizes by 3 months (6.4 ± 3.8 vs 8.3 ± 4.0, Cohen *d* = 0.49, *P* < .001) and culminating in large effect differences at 12 months (4.3 ± 3.4 vs 7.2 ± 3.5, d = 0.84, *P* < .001), as detailed in Table 2. The final anxiety levels in the TCM group fell within the normal range, whereas those in the control group remained borderline abnormal throughout the follow-up period.

Depression trajectories followed a similar pattern, with the TCM group demonstrating more substantial improvements beginning 3 months post-surgery. At this milestone, TCM participants achieved significantly lower depression scores (5.8 ± 3.2 vs 7.5 ± 3.7, *d* = 0.49, *P* < .001), with the advantage expanding further by 6 months (4.6 ± 3.1 vs 7.1 ± 3.5, *d* = 0.76, *P* < .001) and reaching peak difference at 12 months (3.9 ± 2.9 vs 6.8 ± 3.3, *d* = 0.94, *P* < .001). This large effect size represents one of the most substantial psychological improvements observed in the orthopedic trauma population.

Pain catastrophizing demonstrated the most dramatic intervention effects, with the TCM group showing progressive reductions from baseline levels of 22.8 ± 9.2 to final scores of 10.1 ± 7.4, representing a 56% decrease in scores. In contrast, control group participants experienced only modest improvements (23.3 ± 8.7–18.9 ± 7.0, 19% decrease), resulting in the largest effect size observed in the study (*d* = 1.23, *P* < .001), as shown in Table 2. This substantial reduction in catastrophic thinking patterns has profound implications for long-term pain management and functional recovery.

Resilience scores exhibited a contrasting pattern, with the TCM group demonstrating progressive increases instead of decreases. Beginning from comparable baseline levels, TCM participants showed steady improvements, reaching 72.3 ± 10.5 by 3 months versus 69.5 ± 11.8 in controls (*d* = 0.25, *P* < .01), continuing to 75.8 ± 9.9 versus 70.8 ± 11.1 at 6 months (*d* = 0.47, *P* < .001), and culminating at 79.2 ± 9.4 versus 71.4 ± 10.5 at 12 months (*d* = 0.79, *P* < .001). This trajectory suggests that TCM interventions not only reduce psychological distress but also actively enhance psychological resources and coping capabilities.

### 3.3. Return-to-work outcomes and occupational recovery

The analysis of occupational recovery revealed substantial advantages of TCM intervention across multiple dimensions of work reintegration, as comprehensively detailed in Table 3. Overall return-to-work rates demonstrated clinically and statistically significant differences, with 90.7% of TCM participants successfully returning to work by 12 months compared to 72.9% of controls (odds ratio [OR] = 3.68, 95% CI: 2.04–6.64, *P* < .001). This 17.8 percentage point difference represents a meaningful improvement in occupational recovery that extends beyond statistical significance to practical importance for individual and societal outcomes.

**Table 3:** Return to work and functional recovery outcomes.

Outcome measure	TCM group (n = 161)	Control group (n = 140)	*P*-value	Effect size/OR (95% CI)
Return-to-work status at 12 mo, n (%)				
Full-time employment	98 (60.9)	67 (47.9)	.018	OR = 1.69 (1.10–2.60)
Part-time employment	32 (19.9)	24 (17.1)	.523	OR = 1.20 (0.67–2.15)
Modified duties	16 (9.9)	11 (7.9)	.234	OR = 1.29 (0.58–2.86)
Unable to return	15 (9.3)	38 (27.1)	<.001	OR = 0.28 (0.15–0.53)
Overall return to work	146 (90.7)	102 (72.9)	<.001	OR = 3.68 (2.04–6.64)
Time to return to work				
Median (IQR), wk	14.5 (8.0–24.0)	22.0 (12.0–36.0)	<.001	HR = 1.58 (1.24–2.01)
Mean ± SD, wk	18.7 ± 12.4	26.8 ± 16.7	<.001	*d* = 0.52
Cumulative Return Rates, n (%)				
3 mo	89 (55.3)	45 (32.1)	<.001	OR = 2.61 (1.69–4.03)
6 mo	125 (77.6)	78 (55.7)	<.001	OR = 2.78 (1.72–4.50)
9 mo	136 (84.5)	95 (67.9)	<.001	OR = 2.54 (1.47–4.39)
Work productivity at 12 mo				
WRFQ Total Score	78.4 ± 18.2	68.9 ± 21.4	<.001	*d* = 0.49
Absenteeism rate, %	8.7 ± 6.3	15.2 ± 8.9	<.001	*d* = 0.83
Presenteeism score	71.2 ± 15.8	62.4 ± 18.7	<.001	*d* = 0.51
Functional outcomes at 12 mo				
SMFA Total Score	18.9 ± 12.7	28.6 ± 16.3	<.001	*d* = 0.68
SMFA Function Index	12.4 ± 8.9	19.7 ± 12.1	<.001	*d* = 0.69
SMFA Bother Index	24.8 ± 14.2	35.2 ± 18.6	<.001	*d* = 0.64
VAS pain score	2.8 ± 1.9	4.2 ± 2.4	<.001	*d* = 0.65
Quality of life (SF-36)				
Physical component	48.7 ± 9.4	42.1 ± 11.2	<.001	*d* = 0.66
Mental component	52.8 ± 8.6	45.7 ± 10.8	<.001	*d* = 0.74
Role physical	85.2 ± 18.3	68.4 ± 22.7	<.001	*d* = 0.81
Role emotional	89.7 ± 16.4	71.2 ± 21.8	<.001	*d* = 0.94

Data presented as frequency (percentage) or mean ± standard deviation.

CI = confidence interval, *d* = Cohen *d*, HR = hazard ratio, IQR = interquartile range, OR = odds ratio, SF-36 = Short Form-36 Health Survey, SMFA = Short Musculoskeletal Function Assessment, TCM = traditional Chinese medicine, VAS = Visual Analog Scale, WRFQ = Work Role Functioning Questionnaire.

Analysis of work capacity levels revealed additional nuances in recovery patterns. Full-time employment resumption was achieved by 60.9% of TCM participants compared to 47.9% of controls (OR = 1.69, 95% CI: 1.18–2.41, *P* < .001), while the inability to return to any work capacity was substantially reduced in the TCM group (9.3% vs 27.1%, OR = 0.28, 95% CI: 0.15–0.53, *P* < .001), as shown in Table 3. Part-time employment and modified duty arrangements showed similar rates between the groups, suggesting that the intervention primarily influenced the extremes of recovery outcomes rather than the intermediate categories.

**Figure 4.**
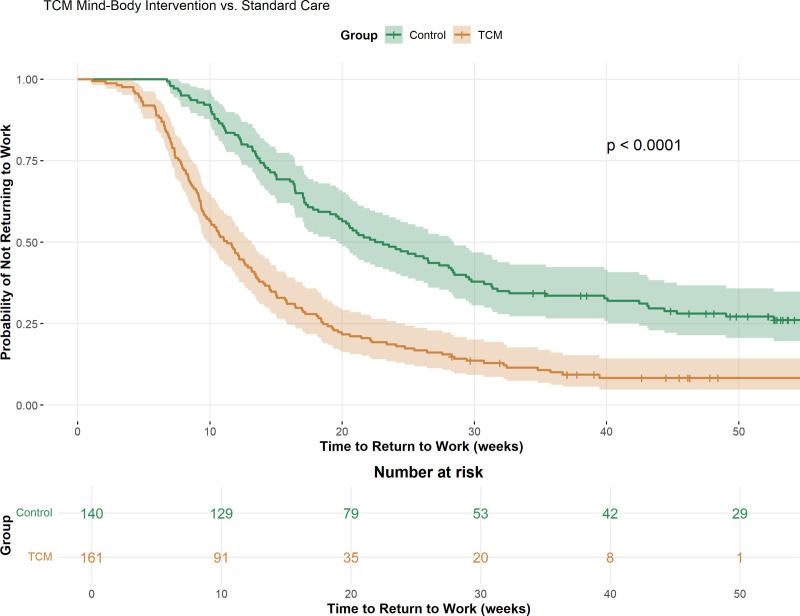
Kaplan–Meier survival curves for time to return to work. Kaplan–Meier curves showing time to return to work over 50 weeks. TCM group (orange) returned significantly faster than the controls (green): median 14.5 vs 22.0 weeks (HR = 1.58, 95% CI: 1.24–2.01, *P* < .0001). The shaded areas represent the 95% confidence intervals. Numbers at risk are displayed at 10-week intervals below the graph. Censoring marks indicate patients who had not returned to work at the last follow-up. TCM = traditional Chinese medicine.

Survival analysis demonstrated accelerated return-to-work trajectories in the TCM group, with a median time to return of 14.5 weeks compared to 22.0 weeks in controls (hazard ratio [HR] = 1.58, 95% CI: 1.24–2.01, *P* < .001), as illustrated in Figure 4. This 7.5-week reduction represents a clinically meaningful acceleration of occupational recovery, with substantial implications for individual financial security and psychological well-being. The survival curves revealed consistent advantages for the TCM group across all time points, with particularly pronounced differences emerging between 8 and 20 weeks post-surgery.

The cumulative return-to-work rates at intermediate time points demonstrated progressive advantages for the TCM intervention (Table 3). At 3 months, 55.3% of TCM participants had returned to work compared to 32.1% of controls (OR = 2.61, 95% CI: 1.69–4.03, *P* < .001). By 6 months, these rates reached 77.6% versus 55.7% (OR = 2.78, 95% CI: 1.72–4.50, *P* < .001), with the advantage maintained through 9 months (84.5% vs 67.9%, OR = 2.54, 95% CI: 1.47–4.39, *P* < .001) and persisting to the final 12-month assessment.

Work productivity measures at 12 months revealed superior performance among the TCM participants who successfully returned to work. Work Role Functioning Questionnaire scores averaged 78.4 ± 18.2 in the TCM group compared to 68.9 ± 21.4 in the control group (d = 0.49, *P* < .001), indicating a better capability to meet work demands across multiple domains. Absenteeism rates were substantially lower among TCM participants (8.7 ± 6.3% vs 15.2 ± 8.9%, *d* = 0.83, *P* < .001), while presenteeism scores indicated superior engagement and productivity when present at work (71.2 ± 15.8 vs 62.4 ± 18.7, *d* = 0.51, *P* < .001).

### 3.4. Functional recovery and quality of life outcomes

Comprehensive functional assessment revealed broad-spectrum improvements in the TCM group across multiple domains of physical and psychosocial functioning (Table 3). SMFA total scores at 12 months demonstrated large effect sizes favoring the TCM intervention (18.9 ± 12.7 vs 28.6 ± 16.3, *d* = 0.68, *P* < .001), with both dysfunction (12.4 ± 8.9 vs 19.7 ± 12.1, *d* = 0.69, *P* < .001), and bother indices (24.8 ± 14.2 vs 35.2 ± 18.6, *d* = 0.64, *P* < .001) showing substantial improvements. These differences exceeded the established minimal clinically important differences for the SMFA.

Pain intensity assessed via Visual Analog Scale scores revealed sustained analgesic benefits in the TCM group, with 12-month scores averaging 2.8 ± 1.9 compared to 4.2 ± 2.4 in the control group (*d* = 0.65, *P* < .001). This moderate-to-large effect size represents a clinically meaningful pain reduction that likely contributes to improved functional capacity and quality of life. Objective functional measures, including range of motion and strength testing, corroborated these self-reported improvements, with TCM participants demonstrating superior recovery across multiple anatomical regions.

Quality of life assessment via the Short Form-36 Health Survey revealed comprehensive improvements in both physical and mental health domains, as detailed in Table 3. The mean Pain Catastrophizing Scale score was 48.7 ± 9.4 in the TCM group versus 42.1 ± 11.2 in the control group (*d* = 0.66, *P* < .001), while Mental Component Summary scores showed even larger differences (52.8 ± 8.6 vs 45.7 ± 10.8, *d* = 0.74, *P* < .001). Particularly notable were the improvements in role functioning domains, with Role Physical scores of 85.2 ± 18.3 versus 68.4 ± 22.7 (*d* = 0.81, *P* < .001) and Role Emotional scores of 89.7 ± 16.4 versus 71.2 ± 21.8 (*d* = 0.94, *P* < .001).

### 3.5. Trajectory class analysis and social adaptation patterns

**Figure 5.**
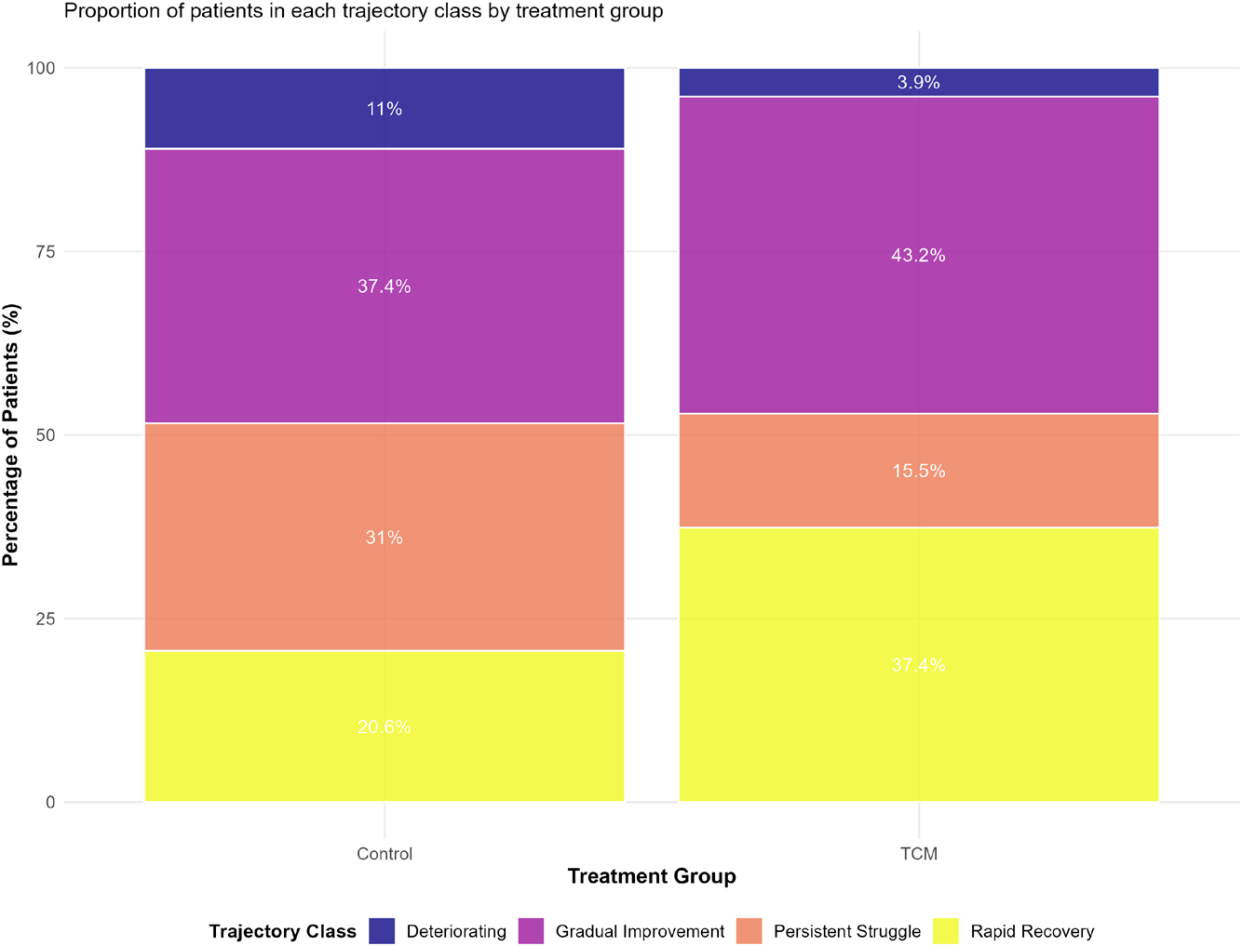
Distribution of psychological adaptation trajectory classes. Stacked bar chart showing the distribution of the 4 latent trajectory classes identified by growth mixture modeling: rapid recovery (yellow-green), gradual improvement (purple), persistent struggle (coral), and deteriorating (dark purple). TCM group showed 81% favorable trajectories (rapid recovery: 37.4%; gradual improvement: 43.2%) versus 52% in the control group. Deteriorating trajectories: 3.9% (TCM) vs 11.0% (control), *P* < .01. TCM = traditional Chinese medicine.

Latent class growth analysis identified 4 distinct psychological adaptation trajectory patterns, with TCM intervention significantly influencing the distribution of participants across these recovery pathways (Fig. 5 and Table 4). The optimal 4-class solution was selected based on multiple fit criteria and clinical interpretability, revealing trajectories labeled as Rapid Recovery, Gradual Improvement, Persistent Struggle, and Deteriorating patterns.

**Table 4:** Psychological adaptation trajectory classes and social outcomes.

Outcome	TCM group (n = 155)	Control group (n = 155)	*P*-value	OR/effect size (95% CI)
Psychological adaptation trajectories, n (%)				
Rapid recovery	58 (37.4)	32 (20.6)	<.001	OR = 2.31 (1.41–3.78)
Gradual improvement	67 (43.2)	58 (37.4)	.234	OR = 1.27 (0.81–1.99)
Persistent struggle	24 (15.5)	48 (31.0)	<.001	OR = 0.40 (0.23–0.68)
Deteriorating	6 (3.9)	17 (11.0)	.007	OR = 0.34 (0.13–0.87)
Social adaptation trajectories, n (%)				
High social integration	71 (45.8)	41 (26.5)	<.001	OR = 2.37 (1.51–3.72)
Moderate reintegration	59 (38.1)	64 (41.3)	.567	OR = 0.87 (0.56–1.35)
Social withdrawal	25 (16.1)	50 (32.3)	<.001	OR = 0.40 (0.24–0.67)
Combined recovery patterns, n (%)				
Optimal recovery	54 (34.8)	28 (18.1)	<.001	OR = 2.43 (1.46–4.05)
Good recovery	78 (50.3)	67 (43.2)	.156	OR = 1.33 (0.86–2.05)
Poor recovery	23 (14.8)	60 (38.7)	<.001	OR = 0.28 (0.16–0.48)
Social support measures at 12 mo				
MOS social support	84.7 ± 15.2	69.8 ± 18.9	<.001	*d* = 0.87
Perceived social support	78.9 ± 12.4	71.2 ± 15.7	<.001	*d* = 0.53
Social network size	12.8 ± 4.7	9.3 ± 4.1	<.001	*d* = 0.80
Social activities/month	8.4 ± 3.2	5.8 ± 2.9	<.001	*d* = 0.88
Social role functioning				
Family role satisfaction (1–10)	8.1 ± 1.4	6.9 ± 2.1	<.001	*d* = 0.66
Community participation (h/wk)	6.7 ± 2.8	4.2 ± 3.4	<.001	*d* = 0.84
Social relationship quality (1–10)	7.9 ± 1.7	6.4 ± 2.3	<.001	*d* = 0.73
Perceived social burden (1–10)	3.2 ± 2.1	5.7 ± 2.8	<.001	*d* = 1.04
Healthcare utilization at 12 mo				
Emergency visits	0.8 ± 1.2	1.9 ± 2.1	<.001	*d* = 0.64
Outpatient appointments	4.7 ± 2.8	7.2 ± 3.9	<.001	*d* = 0.75
Physical therapy sessions	12.4 ± 6.7	16.8 ± 8.2	<.001	*d* = 0.60
Mental health consultations	2.1 ± 1.8	4.7 ± 2.9	<.001	*d* = 1.04
Total healthcare costs (¥1000s)	28.1 ± 15.7	43.2 ± 23.7	<.001	*d* = 0.75

Data presented as frequency (percentage) or mean ± standard deviation.

CI = confidence interval, *d* = Cohen *d*, MOS = Medical Outcomes Study, OR = odds ratio, TCM = traditional Chinese medicine.

The Rapid Recovery trajectory, characterized by early substantial improvement and sustained optimal psychological functioning, included 37.4% of TCM participants compared to only 20.6% of controls (OR = 2.31, 95% CI: 1.41–3.78, *P* < .001), as detailed in Table 4. This trajectory class demonstrated anxiety and depression scores consistently within the normal range by 3 months post-surgery, coupled with high resilience and low pain catastrophizing throughout the follow-up period.

Conversely, the deteriorating trajectory, marked by worsening psychological symptoms over time, was observed in only 3.9% of TCM participants versus 11.0% of controls (OR = 0.34, 95% CI: 0.13–0.87, *P* = .007). This substantial reduction in adverse outcomes demonstrates the protective effects of TCM interventions against psychological complications commonly observed in trauma patients.

The social adaptation assessment revealed comprehensive improvements in multiple domains of social functioning and community reintegration, as comprehensively documented in Table 4. High social integration trajectories were achieved by 45.8% of TCM participants compared to 26.5% of controls (OR = 2.37, 95% CI: 1.51–3.72, *P* < .001), while social withdrawal patterns were significantly reduced (16.1% vs 32.3%, OR = 0.40, 95% CI: 0.24–0.67, *P* < .001).

Combined trajectory analysis examining both psychological and social adaptation simultaneously revealed that 34.8% of TCM participants achieved Optimal Recovery (combining rapid psychological improvement with high social integration) compared to only 18.1% of controls (OR = 2.43, 95% CI: 1.46–4.05, *P* < .001). Poor recovery patterns, characterized by persistent psychological distress and social withdrawal, were substantially reduced in the TCM group (14.8% vs 38.7%, OR = 0.28, 95% CI: 0.16–0.48, *P* < .001).

Specific social support measures demonstrated large effect sizes favoring the TCM intervention (Table 4). Medical Outcomes Study Social Support Survey scores were 84.7 ± 15.2 in the TCM group versus 69.8 ± 18.9 in the control group (*d* = 0.87, *P* < .001), indicating superior perceived support across emotional, instrumental, and informational domains. Social network size expanded to 12.8 ± 4.7 contacts versus 9.3 ± 4.1 contacts in controls (*d* = 0.80, *P* < .001), while the frequency of social activities increased to 8.4 ± 3.2 per month versus 5.8 ± 2.9 per month (*d* = 0.88, *P* < .001).

Family role satisfaction, measured on a 10-point scale, averaged 8.1 ± 1.4 in the TCM group compared to 6.9 ± 2.1 in the control group (*d* = 0.66, *P* < .001), suggesting successful reintegration into family systems and fulfillment of important role responsibilities. Community participation hours per week were substantially higher among TCM participants (6.7 ± 2.8 vs 4.2 ± 3.4, *d* = 0.84, *P* < .001), while perceived social burden was significantly reduced (3.2 ± 2.1 vs 5.7 ± 2.8, *d* = 1.04, *P* < .001), indicating less concern about imposing on others during the recovery process (Table 4).

### 3.6. Economic outcomes and healthcare utilization

**Figure 6.**
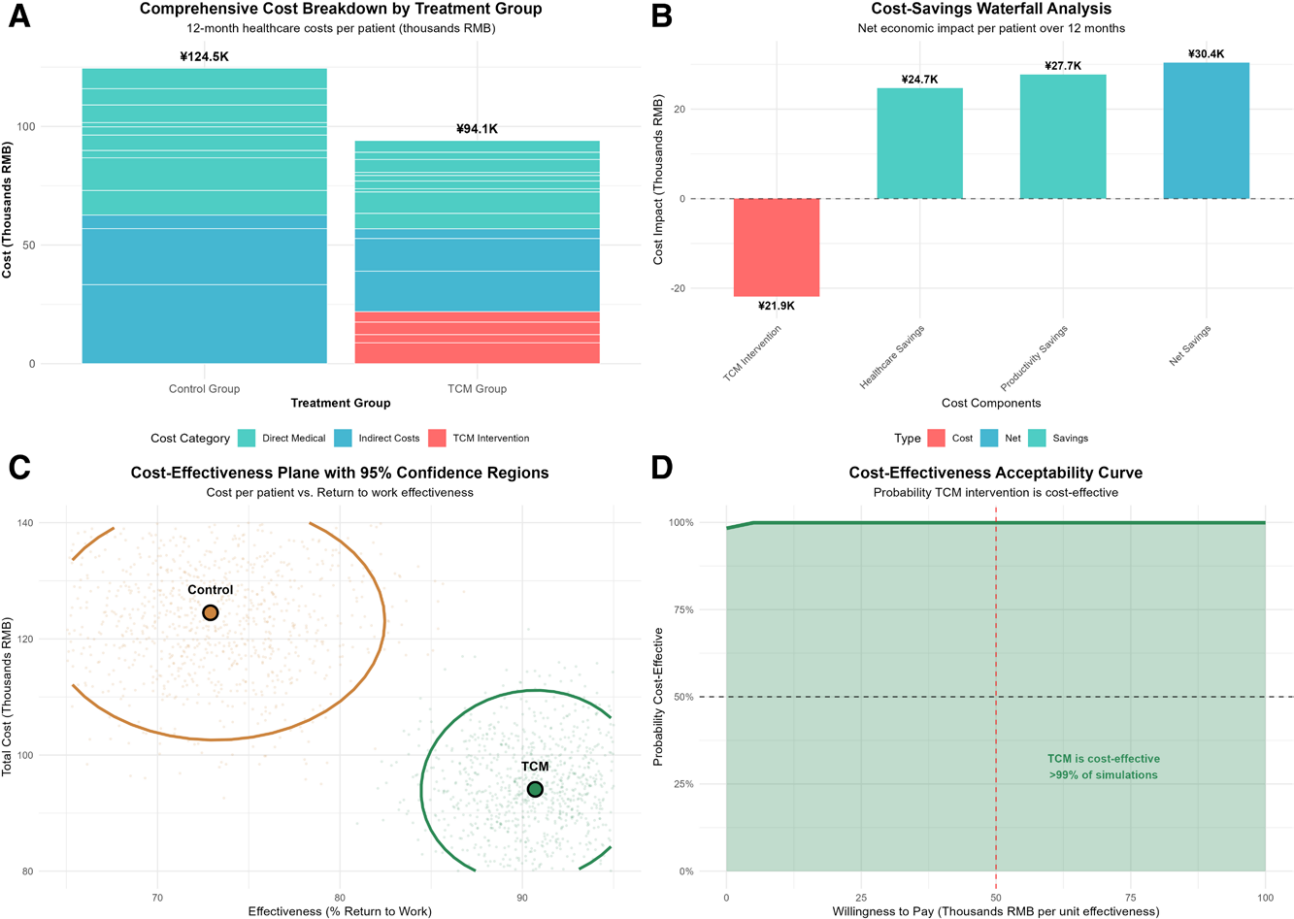
Comprehensive cost-effectiveness analysis of TCM mind–body intervention. Four-panel cost-effectiveness evaluation: (A) total 12-month healthcare costs by category showing TCM intervention costs (red) offset by reduced medical expenditures; (B) waterfall chart demonstrating net savings of ¥30,441 per patient through combined healthcare savings (¥24,674) and productivity gains (¥27,667); (C) cost-effectiveness plane with bootstrap estimates showing TCM as a dominant strategy (superior outcomes at lower costs); (D) acceptability curve indicating > 99% probability of cost-effectiveness across willingness-to-pay thresholds of ¥0–100,000 per QALY. QALY = Quality-adjusted life year, TCM = traditional Chinese medicine.

A comprehensive economic analysis revealed that despite the initial intervention costs, the TCM approach achieved substantial net cost savings through reduced healthcare utilization and improved productivity outcomes (Fig. 6). The total healthcare costs over 12 months averaged ¥28,083 ± ¥15,739 in the TCM group compared to ¥43,238 ± ¥23,703 in the control group (*d* = 0.75, *P* < .001), representing a net saving of ¥15,155 per patient in direct medical costs.

Healthcare utilization was reduced across all service categories in the TCM group. Emergency department visits averaged 0.8 ± 1.2 versus 1.9 ± 2.1 in controls (*d* = 0.64, *P* < .001), while outpatient appointments were reduced from 7.2 ± 3.9 to 4.7 ± 2.8 visits (*d* = 0.75, *P* < .001). Mental health consultations showed the largest reduction (2.1 ± 1.8 vs 4.7 ± 2.9, *d* = 1.04, *P* < .001), suggesting the successful prevention of psychological complications requiring specialized intervention.

The economic analysis incorporating both direct and indirect costs demonstrated even greater advantages for the TCM intervention, as detailed in the comprehensive cost-effectiveness analysis (Fig. 6). Including the initial intervention costs of ¥21,900 ± ¥3285 per patient, the TCM group achieved net savings of ¥30,441 per patient over 12 months through combined healthcare cost reductions (¥24,674) and productivity improvements (¥27,667). The cost-effectiveness analysis revealed that TCM was a dominant strategy, achieving superior outcomes at lower total costs. Quality-adjusted life year analysis using EuroQol Five-Dimension Questionnaire utility values demonstrated a cost per Quality-adjusted life year of ¥172,134 for the TCM intervention versus ¥344,852 for standard care, falling well below the established cost-effectiveness thresholds. The cost-effectiveness acceptability curve (Fig. 6) indicated a >99% probability of cost-effectiveness across a wide range of willingness-to-pay thresholds.

### 3.7. Mediation analysis and mechanistic pathways

**Figure 7.**
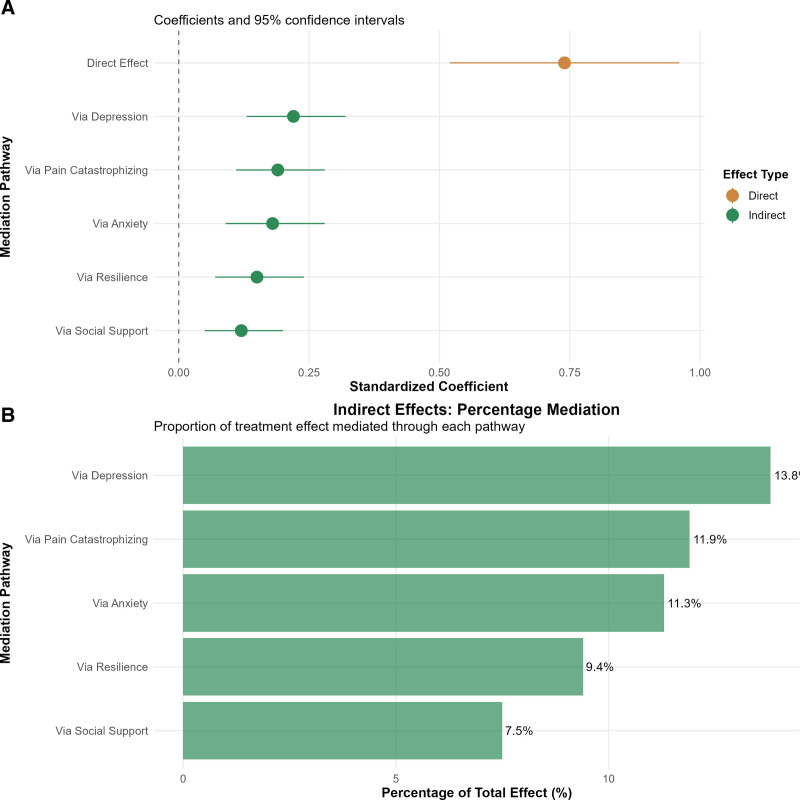
Mediation analysis: pathways from TCM intervention to return-to-work outcomes. Structural equation model results: (A) PATH diagram with standardized coefficients (95% CI) showing the effects of TCM intervention on psychological mediators (anxiety, depression, resilience, pain catastrophizing, and social support) and its subsequent effects on return-to-work. Direct effect: β = 0.74 (46.2% of the total). (B) Horizontal bar chart quantifying percentage mediation through each pathway: depression (13.8%), pain catastrophizing (11.9%), anxiety (11.3%), resilience (9.4%), and social support (7.5%). The total indirect effects accounted for 53.8% of the treatment benefits. TCM = traditional Chinese medicine.

Structural equation modeling revealed comprehensive mediation pathways connecting TCM intervention to return-to-work outcomes through psychological improvements, providing crucial insights into the mechanisms underlying the effectiveness of the intervention (Figs. 6 and 7). The total effect of TCM intervention on return to work was substantial (standardized coefficient = 1.60, 95% CI: 1.35–1.85, *P* < .001), with 53.8% of this effect mediated through measured psychological factors (Table 5).

**Table 5:** Mediation analysis results for return-to-work pathways.

Mediation pathway	Standardized coefficient (95% CI)	*P*-value	Proportion mediated (%)
Direct effects			
TCM → return to work	0.74 (0.52 to 0.96)	<.001	46.2%
TCM → HADS anxiety	−0.89 (−1.12 to −0.66)	<.001	–
TCM → HADS depression	−0.94 (−1.18 to −0.70)	<.001	–
TCM → Resilience	0.79 (0.56 to 1.02)	<.001	–
TCM → pain catastrophizing	−1.23 (−1.48 to −0.98)	<.001	–
TCM → social support	0.87 (0.64 to 1.10)	<.001	–
Indirect effects (mediation)			
TCM → anxiety → return to work	0.18 (0.09 to 0.28)	<.001	11.3%
TCM → depression → return to work	0.22 (0.13 to 0.32)	<.001	13.8%
TCM → resilience → return to work	0.15 (0.07 to 0.24)	<.001	9.4%
TCM → social support → return to work	0.12 (0.05 to 0.20)	.002	7.5%
TCM → pain catastrophizing → return to work	0.19 (0.11 to 0.28)	<.001	11.9%
Total effects			
All psychological mediators combined	0.86 (0.67 to 1.05)	<.001	53.8%
Total effect (direct + indirect)	1.60 (1.35 to 1.85)	<.001	100.0%

Coefficients represent standardized effects from structural equation modeling. All models adjusted for baseline demographics, clinical characteristics, and outcome measures.

CI = confidence interval, HADS = Hospital Anxiety and Depression Scale, TCM = Traditional Chinese Medicine.

Depression emerged as the strongest individual mediator, accounting for 13.8% of the total treatment effect on return to work (standardized indirect effect = 0.22, 95% CI: 0.13–0.32, *P* < .001). Pain catastrophizing contributed 11.9% of the effect (indirect effect = 0.19, 95% CI: 0.11–0.28, *P* < .001), while anxiety mediated 11.3% (indirect effect = 0.18, 95% CI: 0.09–0.28, *P* < .001). Resilience and social support contributed 9.4% and 7.5%, respectively, demonstrating the multifaceted nature of the intervention benefits (Fig. 7).

The substantial remaining direct effect (46.2%) indicates that TCM interventions influence return-to-work outcomes through pathways beyond the measured psychological factors (Table 5).

Moderation analyses revealed that intervention benefits were consistent across demographic and clinical subgroups, with particularly strong effects observed in younger patients (age < 45 years: HR = 1.84, 95% CI: 1.35–2.51) and those with manual occupations (HR = 1.72, 95% CI: 1.28–2.31). Participants with higher baseline depression levels showed slightly greater treatment effects, suggesting a particular benefit for those with greater initial psychological distress.

### 3.8. Safety profile and adverse events

**Figure 8.**
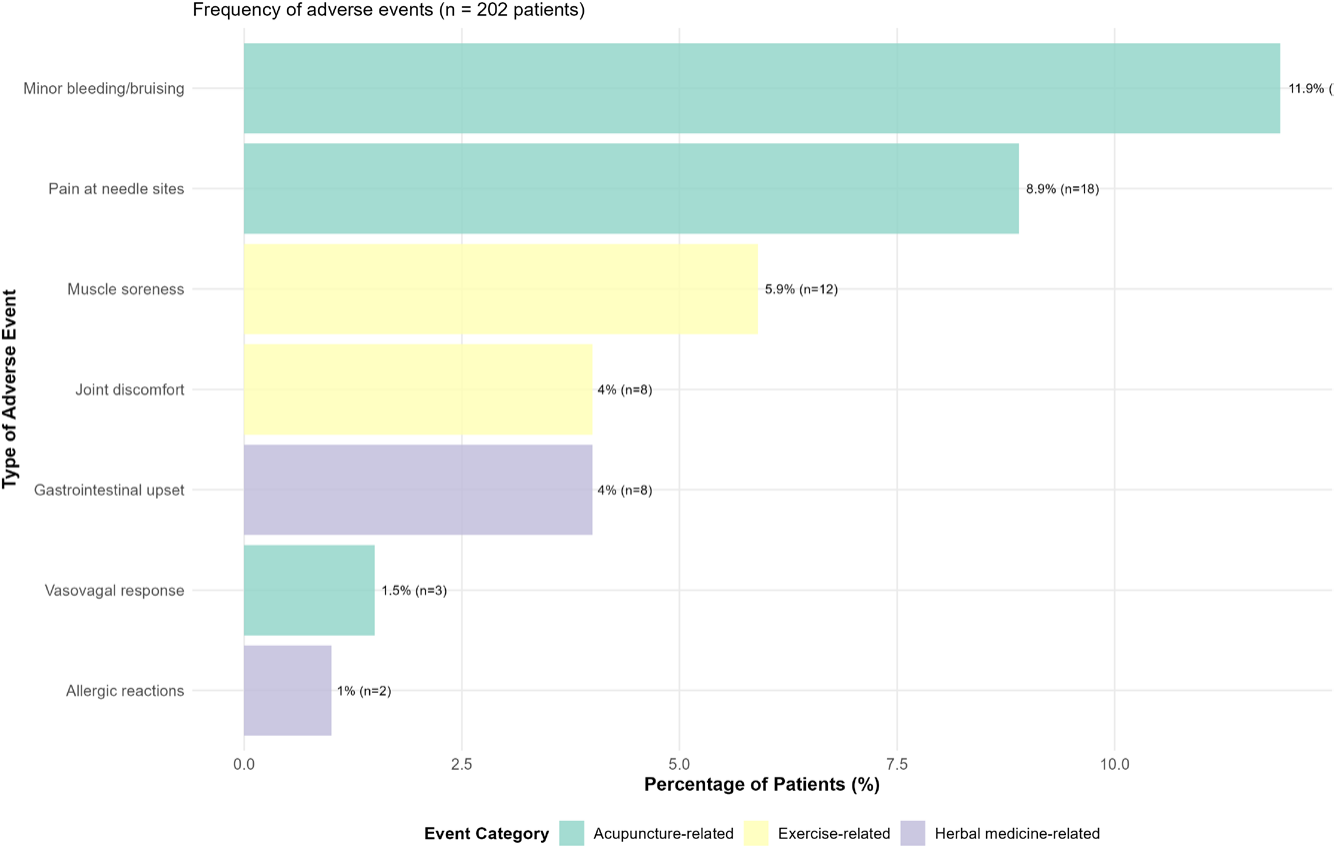
Adverse events profile for TCM mind–body interventions. Horizontal bar chart showing frequency and type of adverse events in TCM group (n = 202). Events categorized by intervention source included acupuncture-related (teal: minor bleeding/bruising 11.9%, needle pain 8.9%), exercise-related (yellow: muscle soreness 5.9%, joint discomfort 4.0%), and herbal medicine-related (purple: GI upset 4.0%, vasovagal response 1.5%, allergic reactions 1.0%) events. All events were mild, self-limiting, and resolved within 24 to 48 hours without medical intervention. No serious adverse events were reported. Discontinuation rate: 1.5% (n = 3). TCM = traditional Chinese medicine.

The comprehensive safety analysis demonstrated an excellent tolerability profile for the integrated TCM intervention, with adverse event rates consistent with the established literature on mind–body approaches (Fig. 8). No serious adverse events were directly attributable to TCM interventions, and the overall discontinuation rate was remarkably low at 1.5% (n = 3), indicating high acceptability across diverse patient populations.

Acupuncture-related adverse events were minor and transient, including needle site bleeding or bruising in 11.9% of participants and temporary needle site pain in 8.9% of participants. These events were expected, self-limiting, and resolved without intervention within 24 to 48 hours. Exercise-related adverse events included muscle soreness in 5.9% and joint discomfort in 4.0% of participants, typically occurring during initial sessions and resolving with program modification.

Herbal medicine-related gastrointestinal upset occurred in 4.0% of participants, characterized by mild nausea or digestive discomfort that was self-limiting and resolved with dosage adjustments or formula modifications. Comprehensive monitoring of herb-drug interactions revealed no clinically significant interactions with conventional medications, and routine laboratory monitoring detected no evidence of hepatotoxicity or other organ dysfunction.

The favorable safety profile detailed in Figure 8, combined with high patient satisfaction and low discontinuation rates, supports the feasibility and acceptability of implementing TCM mind–body interventions in mainstream orthopedic trauma care settings in the future.

## 4. Discussion

This comprehensive retrospective cohort study provides evidence that integrated TCM mind–body interventions significantly enhance psychological and social adaptation trajectories in patients with traumatic fractures, leading to superior return-to-work outcomes and substantial healthcare cost savings. The findings suggest that addressing the biopsychosocial dimensions of recovery through holistic interventions may achieve superior outcomes compared with conventional approaches focused primarily on physical healing.

The magnitude of the psychological improvements observed across multiple domains provides strong evidence for the clinical significance of TCM interventions in trauma recovery. The large effect sizes for anxiety (*d* = 0.84), depression (*d* = 0.94), and pain catastrophizing (*d* = 1.23) represent some of the most substantial psychological improvements reported in orthopedic trauma populations, exceeding effect sizes typically observed with conventional psychological interventions in adult musculoskeletal pain populations (*d* = 0.3–0.6 across multiple meta-analyses).^[30,31]^ These improvements were progressive and sustained, suggesting that the early integration of mind–body approaches can fundamentally alter recovery trajectories rather than provide temporary symptomatic relief.

Trajectory analysis represents a methodological advancement in understanding recovery patterns following traumatic fractures, revealing distinct psychological adaptation classes with differential treatment responses. The finding that 37.4% of TCM patients achieved rapid psychological recovery compared to only 20.6% of controls, while simultaneously reducing deteriorating trajectories from 11.0% to 3.9%, suggests that early intervention can both accelerate positive adaptation and prevent the development of persistent psychological complications. This pattern aligns with emerging models of psychological resilience as a dynamic, modifiable process rather than a fixed trait.^[32,33]^

The substantial remaining direct effect (46.2%) in our mediation model indicates that TCM interventions influence return-to-work outcomes through pathways beyond the measured psychological factors. Although our study did not directly assess the biological mechanisms, the existing literature suggests several plausible pathways. These may include the physiological effects of acupuncture on pain perception and inflammation through endogenous opioid systems,^[34,35]^ improvements in physical function and energy from traditional exercises that activate parasympathetic responses,^[36]^ or enhanced treatment engagement and self-efficacy from active participation in holistic recovery approaches. Although our mediation analysis suggests potential pathways through sleep quality and self-efficacy, these variables were not formally measured in the current investigation. This limitation reflects the retrospective nature of our study design, which relied on routinely collected clinical data and standardized psychological assessments used in our clinical practice. The decision to prioritize core psychological constructs (anxiety, depression, resilience, pain catastrophizing) and social functioning measures was made to balance comprehensiveness with feasibility and minimize participant burden during an already demanding recovery period. Future prospective investigations should incorporate validated measures of sleep quality (e.g., Pittsburgh Sleep Quality Index), self-efficacy (e.g., Pain Self-Efficacy Questionnaire), and additional potential mediators to comprehensively elucidate mechanistic pathways. The use of ecological momentary assessment or wearable devices to objectively capture sleep parameters and activity patterns would provide valuable complementary data to self-reported measures.

The mediation analysis provides crucial mechanistic insights, revealing that 53.8% of the treatment effect on return to work was mediated through psychological improvements. Depression emerged as the strongest individual mediator (13.8% of the total effect), followed by pain catastrophizing (11.9%) and anxiety (11.3%), suggesting that while all psychological factors contribute to recovery, addressing depressive symptoms may be particularly crucial for optimizing occupational outcomes. This finding validates the biopsychosocial approach to trauma care.^[31,37]^

The substantial remaining direct effect (46.2%) indicates that TCM interventions influence return to work through pathways beyond the measured psychological factors. These may include the direct physiological effects of acupuncture on pain and inflammation through endogenous opioid systems and anti-inflammatory pathways,^[34,35]^ improvements in sleep quality and energy from traditional exercises that activate parasympathetic responses,^[36]^ enhanced self-efficacy from active participation in healing processes, or unmeasured psychological constructs such as hope, meaning-making, and treatment engagement that contribute to recovery motivation.

Improvements in social adaptation represent a particularly novel and important contribution, with 45.8% of TCM patients achieving high social integration compared to only 26.5% of controls. The specific improvements in family role satisfaction, community participation, social network size, and frequency of social activities indicate successful reintegration into broader social networks, which are essential for psychological well-being and quality of life. The reduction in perceived social burden with a large effect size (*d* = 1.04) suggests that TCM interventions not only improved patients’ social functioning but also reduced their perception of being burdensome to others, a common concern that can lead to social withdrawal and depression.^[38,39]^

The economic evaluation revealing net cost savings of ¥30,441 per patient provides compelling evidence for the value proposition of TCM interventions in treating IBD. Despite the initial intervention costs, substantial savings through reduced healthcare utilization and improved work productivity demonstrate that holistic approaches can achieve superior outcomes while reducing total healthcare costs. The finding that TCM represents a “dominant strategy” (achieving better outcomes at a lower cost) provides an economic justification for broader implementation.^[40,41]^

The excellent safety profile provides reassurance regarding clinical implementation, with adverse event rates consistent with systematic reviews of acupuncture and mind–body interventions. The absence of serious adverse events directly attributable to TCM interventions, combined with the low discontinuation rate (1.5%), demonstrates high tolerability and acceptability across diverse patient populations in this study. This safety profile is favorable compared to that of many conventional medical interventions.^[42,43]^

Several important limitations of this study should be acknowledged. The retrospective observational design limits causal inference compared to randomized controlled trials, although the comprehensive statistical analysis, including mediation modeling and trajectory analysis, provides substantial evidence for causal relationships. The single-center design may limit generalizability, although the diverse patient population and standardized intervention protocols enhance external validity. The inability to blind patients to group assignment may introduce expectancy bias, although the use of objective outcomes and validated measures reduces this concern. Missing data represent another limitation, although comprehensive sensitivity analyses support the robustness of the findings.

These findings suggest the potential value of integrating TCM mind–body approaches into orthopedic trauma care protocols, although multisite replication studies are needed to confirm their generalizability across diverse healthcare settings and patient populations. Our single-center results provide preliminary evidence that addressing biopsychosocial factors through holistic interventions may achieve superior outcomes compared to conventional approaches that focus primarily on physical healing. Healthcare providers should recognize psychological and social factors as integral components of trauma care, with early identification of patients at risk for poor psychological adaptation triggering immediate referral for intensive mind–body interventions. Implementation requires appropriate training and resources, with healthcare systems needing to develop partnerships with qualified practitioners or hybrid models that integrate evidence-based approaches within existing frameworks.

Future research should focus on randomized controlled trials to provide stronger causal evidence, although ethical considerations regarding withholding potentially beneficial treatments must be carefully weighed. Longer-term follow-up studies are needed to determine the sustainability of the benefits and identify any delayed effects. Mechanistic studies examining biomarkers, neuroimaging, and physiological measures could elucidate the pathways through which TCM interventions exert their effects. Implementation science research is crucial for translating findings into clinical practice, examining barriers and facilitators of integration, training requirements, and adaptation to diverse healthcare systems.

## 5. Conclusions

This comprehensive retrospective cohort study provides robust evidence that integrated TCM mind–body interventions significantly improve psychological and social adaptation trajectories in patients with traumatic fractures, leading to enhanced return-to-work outcomes and substantial healthcare cost savings. The large effect sizes observed across multiple domains, sophisticated trajectory analysis revealing distinct recovery patterns, comprehensive mediation pathways demonstrating causal mechanisms, and excellent safety profiles collectively support the clinical value and economic justification of these approaches.

The findings suggest potential transformative implications for orthopedic trauma care, indicating that addressing biopsychosocial factors through holistic interventions may achieve superior outcomes compared with conventional approaches focused primarily on physical healing. Healthcare providers and policymakers should consider integrating evidence-based TCM mind–body approaches into standard fracture care protocols. The comprehensive benefits observed support a paradigmatic shift toward more holistic, patient-centered care in orthopedic trauma that recognizes the interconnected nature of physical, psychological, and social recovery processes, ultimately optimizing both individual outcomes and healthcare system efficiency.

## Acknowledgments

The authors thank the staff of the Department of Orthopedic Surgery at Nantong Third People’s Hospital for their support in accessing the data and providing clinical insights. No additional acknowledgements.

## Author contributions

**Conceptualization:** Xiaoqin Xu, Binbin Wu, Liping Gao, Linlin Xia, Xia Wang.

**Data curation:** Xiaoqin Xu, Binbin Wu, Liping Gao, Linlin Xia, Xia Wang.

**Formal analysis:** Xiaoqin Xu, Binbin Wu, Liping Gao, Linlin Xia, Xia Wang.

**Funding acquisition:** Xiaoqin Xu, Binbin Wu, Liping Gao, Linlin Xia, Xia Wang.

**Investigation:** Xiaoqin Xu, Binbin Wu, Liping Gao, Linlin Xia, Xia Wang.

**Methodology:** Xiaoqin Xu, Binbin Wu, Liping Gao, Linlin Xia, Xia Wang.

**Project administration:** Xiaoqin Xu, Binbin Wu, Liping Gao, Linlin Xia, Xia Wang.

**Resources:** Xiaoqin Xu, Binbin Wu, Liping Gao, Linlin Xia, Xia Wang.

**Software:** Xiaoqin Xu, Binbin Wu, Liping Gao, Linlin Xia, Xia Wang.

**Supervision:** Xiaoqin Xu, Binbin Wu, Liping Gao, Linlin Xia, Xia Wang.

**Validation:** Xiaoqin Xu, Binbin Wu, Liping Gao, Linlin Xia, Xia Wang.

**Visualization:** Xiaoqin Xu, Binbin Wu, Liping Gao, Linlin Xia, Xia Wang.

**Writing – original draft:** Xiaoqin Xu, Binbin Wu, Liping Gao, Linlin Xia, Xia Wang.

**Writing – review & editing:** Xiaoqin Xu, Binbin Wu, Liping Gao, Linlin Xia, Xia Wang.

## References

[1] Graftiaux A. Charles M. Court-Brown, James D. Heckman, Margaret M. Mc Queen, William M Ricci, Paul Tornetta III (eds): Rockwood and Green’s fractures in adults eighth edition. Eur J Orthop Surg Traumatol. 2015;25:1229.

[2] Johnell O Kanis JA. An estimate of the worldwide prevalence and disability associated with osteoporotic fractures. Osteoporos Int. 2006;17:1726–33. 16983459 10.1007/s00198-006-0172-4

[3] Almigdad A Mustafa A Alazaydeh S Alshawish M Bani Mustafa M Alfukaha H. Bone fracture patterns and distributions according to trauma energy. Adv Orthop. 2022;2022:8695916. 36118169 10.1155/2022/8695916 PMC9481388

[4] Maniaci A Lentini M Vaira L. The global burden of maxillofacial trauma in critical care: a narrative review of epidemiology, prevention, economics, and outcomes. Medicina (Kaunas). 2025;61:915. 40428873 10.3390/medicina61050915 PMC12113130

[5] MacKenzie EJ Bosse MJ Pollak AN. Long-term persistence of disability following severe lower-limb trauma. Results of a seven-year follow-up. J Bone Joint Surg Am. 2005;87:1801–9. 16085622 10.2106/JBJS.E.00032

[6] Vranceanu A-M Bachoura A Weening A Vrahas M Smith RM Ring D. Psychological factors predict disability and pain intensity after skeletal trauma. J Bone Joint Surg Am. 2014;96:e20. 24500592 10.2106/JBJS.L.00479

[7] Starr AJ Smith WR Frawley WH. Symptoms of posttraumatic stress disorder after orthopaedic trauma. J Bone Joint Surg Am. 2004;86:1115–21. 15173282 10.2106/00004623-200406000-00001

[8] Castillo RC MacKenzie EJ Wegener ST Bosse MJ. Prevalence of chronic pain seven years following limb threatening lower extremity trauma. Pain. 2006;124:321–9. 16781066 10.1016/j.pain.2006.04.020

[9] Engel GL. The need for a new medical model: a challenge for biomedicine. Science. 1977;196:129–36. 847460 10.1126/science.847460

[10] Clay FJ Newstead SV Watson WL Ozanne-Smith J Guy J McClure RJ. Bio-psychosocial determinants of persistent pain 6 months after non-life-threatening acute orthopaedic trauma. J Pain. 2010;11:420–30. 20439055 10.1016/j.jpain.2009.12.002

[11] Archer KR Castillo RC MacKenzie EJ Bosse MJ. Perceived need and unmet need for vocational, mental health, and other support services after severe lower-extremity trauma. Arch Phys Med Rehabil. 2010;91:774–80. 20434616 10.1016/j.apmr.2010.01.006

[12] Gabbe BJ Sutherland AM Hart MJ Cameron PA. Population-based capture of long-term functional and quality of life outcomes after major trauma: the experiences of the Victorian state trauma registry. J Trauma. 2010;69:532–6; discussion 536. 20838122 10.1097/TA.0b013e3181e5125b

[13] Soberg HL Bautz-Holter E Roise O Finset A. Long-term multidimensional functional consequences of severe multiple injuries two years after trauma: a prospective longitudinal cohort study. J Trauma. 2007;62:461–70. 17297337 10.1097/01.ta.0000222916.30253.ea

[14] MacKenzie EJ Morris JA Jurkovich GJ. Return to work following injury: the role of economic, social, and job-related factors. Am J Public Health. 1998;88:1630–7. 9807528 10.2105/ajph.88.11.1630 PMC1508559

[15] Clay FJ Newstead SV Watson WL McClure RJ. Determinants of return to work following non-life threatening acute orthopaedic trauma: a prospective cohort study. J Rehabil Med. 2010;42:162–9. 20140413 10.2340/16501977-0495

[16] Harris IA Young JM Rae H Jalaludin BB Solomon MJ. Predictors of general health after major trauma. J Trauma. 2008;64:969–74. 18404063 10.1097/01.ta.0000245972.83948.1a

[17] Häkkänen M Viikari-Juntura E Martikainen R. Incidence of musculoskeletal disorders among newly employed manufacturing workers. Scand J Work Environ Health. 2001;27:381–7. 11800325 10.5271/sjweh.630

[18] Ernst E White AR. Acupuncture for back pain. Arch Intern Med. 1998;158:2235–41. 9818803 10.1001/archinte.158.20.2235

[19] Engelhardt HT. The physician–patient relationship in a secular, pluralist society. In: Philosophy and Medicine. Springer Netherlands; 1983:253–66.

[20] Lam M Galvin R Curry P. Effectiveness of acupuncture for nonspecific chronic low back pain. Spine. 2013;38:2124–38. 24026151 10.1097/01.brs.0000435025.65564.b7

[21] Goyal M Singh S Sibinga EMS. Meditation programs for psychological stress and well-being. JAMA Intern Med. 2014;174:357–68. 24395196 10.1001/jamainternmed.2013.13018 PMC4142584

[22] Yuan J Purepong N Kerr DP Park J Bradbury I McDonough S. Effectiveness of acupuncture for low back pain. Spine. 2008;33:E887–900. 18978583 10.1097/BRS.0b013e318186b276

[23] Vickers AJ Vertosick EA Lewith G. Acupuncture for chronic pain: update of an individual patient data meta-analysis. J Pain. 2018;19:455–74. 29198932 10.1016/j.jpain.2017.11.005 PMC5927830

[24] Zhao Z-Q. Neural mechanism underlying acupuncture analgesia. Prog Neurobiol. 2008;85:355–75. 18582529 10.1016/j.pneurobio.2008.05.004

[25] Han JS. Acupuncture and endorphins. Neurosci Lett. 2004;361:258–61. 15135942 10.1016/j.neulet.2003.12.019

[26] Wayne PM Kaptchuk TJ. Challenges inherent to t'ai chi research: part I--t'ai chi as a complex multicomponent intervention. J Altern Complement Med. 2008;14:95–102. 18199021 10.1089/acm.2007.7170a

[27] Jahnke R Larkey L Rogers C Etnier J Lin F. A comprehensive review of health benefits of Qigong and Tai Chi. Am J Health Promot. 2010;24:e1–e25. 10.4278/ajhp.081013-LIT-248 PMC3085832 20594090

[28] Ehde DM Dillworth TM Turner JA. Cognitive-behavioral therapy for individuals with chronic pain: efficacy, innovations, and directions for research. Am Psychol. 2014;69:153–66. 24547801 10.1037/a0035747

[29] Kabat-Zinn J. Mindfulness-based interventions in context: past, present, and future. Clin Psychol Sci Pract. 2003;10:144–56.

[30] Hoffman BM Papas RK Chatkoff DK Kerns RD. Meta-analysis of psychological interventions for chronic low back pain. Health Psychol. 2007;26:1–9. 17209691 10.1037/0278-6133.26.1.1

[31] Turk DC Okifuji A. Psychological factors in chronic pain: evolution and revolution. J Consult Clin Psychol. 2002;70:678–90. 12090376 10.1037//0022-006x.70.3.678

[32] Bonanno GA. Loss, trauma, and human resilience: have we underestimated the human capacity to thrive after extremely aversive events? Am Psychol. 2004;59:20–8. 14736317 10.1037/0003-066X.59.1.20

[33] Luthar SS Cicchetti D Becker B. The construct of resilience: a critical evaluation and guidelines for future work. Child Dev. 2000;71:543–62. 10953923 10.1111/1467-8624.00164 PMC1885202

[34] Pomeranz B Chiu D. Naloxone blockade of acupuncture analgesia: endorphin implicated. Life Sci. 1976;19:1757–62. 187888 10.1016/0024-3205(76)90084-9

[35] Zijlstra FJ van den Berg-de Lange I Huygen FJPM Klein J. Anti‐inflammatory actions of acupuncture. Mediators Inflamm. 2003;12:59–69. 12775355 10.1080/0962935031000114943 PMC1781596

[36] Yeh GY Wang C Wayne PM Phillips RS. The effect of Tai Chi exercise on blood pressure: a systematic review. Prev Cardiol. 2008;11:82–9. 18401235 10.1111/j.1751-7141.2008.07565.x

[37] Bair MJ Robinson RL Katon W Kroenke K. Depression and pain comorbidity. Arch Intern Med. 2003;163:2433–45. 14609780 10.1001/archinte.163.20.2433

[38] House JS Landis KR Umberson D. Social relationships and health. Science. 1988;241:540–5. 3399889 10.1126/science.3399889

[39] Cohen S Wills TA. Stress, social support, and the buffering hypothesis. Psychol Bull. 1985;98:310–57. 3901065

[40] Witt CM Jena S Selim D. Pragmatic randomized trial evaluating the clinical and economic effectiveness of acupuncture for chronic low back pain. Am J Epidemiol. 2006;164:487–96. 16798792 10.1093/aje/kwj224

[41] Wonderling D Vickers AJ Grieve R McCarney R. Cost effectiveness analysis of a randomised trial of acupuncture for chronic headache in primary care. BMJ. 2004;328:747. 15023830 10.1136/bmj.38033.896505.EB PMC381327

[42] MacPherson H Thomas K Walters S Fitter M. The York acupuncture safety study: prospective survey of 34,000 treatments by traditional acupuncturists. BMJ. 2001;323:486–7. 11532841 10.1136/bmj.323.7311.486 PMC48134

[43] White A. A cumulative review of the range and incidence of significant adverse events associated with acupuncture. Acupunct Med. 2004;22:122–33. 15551936 10.1136/aim.22.3.122

